# The Role of Fungi in the Etiology of Multiple Sclerosis

**DOI:** 10.3389/fneur.2017.00535

**Published:** 2017-10-16

**Authors:** Julián Benito-León, Martin Laurence

**Affiliations:** ^1^Department of Neurology, University Hospital “12 de Octubre”, Madrid, Spain; ^2^Department of Medicine, Faculty of Medicine, Complutense University, Madrid, Spain; ^3^Centro de Investigación Biomédica en Red sobre Enfermedades Neurodegenerativas (CIBERNED), Madrid, Spain; ^4^Shipshaw Labs, Montreal, QC, Canada

**Keywords:** multiple sclerosis, fungal infections, dimethyl fumarate, memory B cells, Epstein-Barr virus, HLA-DRB1*15

## Abstract

Multiple sclerosis (MS) is a chronic inflammatory disorder of the central nervous system. Infectious triggers of MS are being actively investigated. Substantial evidence supports the involvement of the Epstein-Barr virus (EBV), though other viruses, bacteria, protists, and fungi are also being considered. Many links between fungi and diseases involving chronic inflammation have been found recently. Evidence linking MS and fungi is reviewed here. The HLA-DRB1*15 allele group is the most important genetic risk factor of MS, and is a risk factor in several other conditions linked to fungal infections. Many biomarkers of MS are consistent with fungal infections, such as IL-17, chitotriosidase, and antibodies against fungi. Dimethyl fumarate (DMF), first used as an industrial fungicide, was recently repurposed to reduce MS symptoms. Its mechanisms of action in MS have not been firmly established. The low risk of MS during childhood and its moderate association with herpes simplex virus type 2 suggest genital exposure to microbes (including fungi) should be investigated as a possible trigger. Molecular and epidemiological evidence support a role for infections such as EBV in MS. Though fungal infections have not been widely studied in MS, many lines of evidence are consistent with a fungal etiology. Future microbiome and serological studies should consider fungi as a possible risk factor for MS, and future clinical studies should consider the effect of fungicides other than DMF on MS symptoms.

## Introduction

Multiple sclerosis (MS) is the most common cause of non-traumatic neurological disability in young adults (1, 2). It is a complex heterogeneous inflammatory disorder characterized by nerve axons in the central nervous system (CNS) losing their myelin sheath (3). Understanding this process appears to be the largest obstacle in developing effective therapies.

The best established risk factors of MS are Epstein-Barr virus (EBV) seropositivity (4–6), major histocompatibility complex class II HLA-DRB1*15 alleles (7), smoking (8), vitamin D deficiency (9), young adulthood (10), and female sex (11). MS is considered an autoimmune disease in which the role of infections is actively debated (4, 12), though proof of autoimmunity is currently lacking.

Numerous infectious agents are suspected of triggering MS, and emerging evidence suggests links between established MS and gut microbiota (12). Though most studies focus on bacteria, fungi may also play an important role (13). Many links between fungi and diseases involving idiopathic inflammation have been found recently. *Candida* in the gut is associated with psoriasis (14, 15) and Crohn’s disease (16, 17). Antibodies against fungi are a risk factor of psoriasis (18, 19), and fungicides improve symptoms (20). Antibodies against fungal mannoproteins are a risk factor of ankylosing spondylitis (21), systemic lupus erythematosus (22, 23), sarcoidosis (24), and Crohn’s disease (25).

Few groups have considered a role for fungi in MS. In 1981, Truss reported the resolution of symptoms in five MS cases following antifungal therapy (26). In 2008, Ramos and colleagues reported finding serum antibodies against *Candida* in seven out of eight MS patients, while finding none in 10 healthy controls (27). In 2010, this association was replicated in a larger case-control study (28), and again in 2013 in a small case-control study that linked anti-*Candida* antibodies in the cerebrospinal fluid (CSF) to MS (29). In this review, we analyze the evidence linking MS to a possible fungal etiology.

## Genetic Susceptibility

The HLA-DRB1*15 allele group is the strongest genetic risk factor of MS (7). Of the three common HLA-DRB1*15 alleles, HLA-DRB1*1501 and HLA-DRB1*1503 are associated with MS, while HLA-DRB1*1502 is not (7). These three alleles are very similar: as compared to HLA-DRB1*1501, HLA-DRB1*1502 substitutes valine for glycine at position 86, and HLA-DRB1*1503 substitutes tyrosine for histidine at position 30 (7). HLA-DRB1*1501 and HLA-DRB1*1503 are two of many alleles forming half of the HLA-DR protein complex, which holds peptides collected by antigen-presenting cells (monocytes, macrophages, dendritic cells, and B cells) for presentation to CD4+ T cells. They thus play a key role in determining which antigens induce a T cell mediated immune response, as well as which antigens warrant naive B cell maturation into memory B cells.

The HLA-DRB1*15 allele group is an important risk factor in many other inflammatory conditions, some of which are suspected of having a fungal etiology (Table 1). Allergic bronchopulmonary aspergillosis (ABPA) is caused by an abnormal immune response against usually benign fungi which are often present in the airways (30). The HLA-DRB1*15 alleles associated with ABPA match those associated with MS: HLA-DRB1*1501 and HLA-DRB1*1503, but not HLA-DRB1*1502 (30, 31). Pulmonary sarcoidosis is suspected of being caused by an immune response to fungal antigens in the airways (32). Granulomatous prostatitis can be caused by fungal infections, though most cases are idiopathic (33); the recent discovery of a fungicidal protein in the prostate (34) suggests a fastidious fungal infection is present in this site (35, 36). Uveitis can be caused by different infectious agents, but in most cases none can be found (37). Uveitis is associated with various idiopathic inflammatory diseases including ankylosing spondylitis, Behçet’s disease, sarcoidosis, and Crohn’s disease (37); it is also associated with MS, and often coincides with MS onset (38). Several studies have linked idiopathic uveitis with fungal infections (Table 1).

**Table 1:** Inflammatory conditions associated with HLA-DRB1*15.

Condition associated with HLA-DRB1*15	Fungal etiology	Antibodies against fungi	Other links with fungi
Allergic bronchopulmonary aspergillosis (30)	Confirmed	*Aspergillus* (39)	Caused by abnormal immune response against *Aspergillus*

Pulmonary sarcoidosis (40)	Suspected	Mannan and beta-glucan (24)	Fungicidal drug itraconazole effective (41)

Granulomatous prostatitis (42)	Suspected	Not tested	Chronic fungal infection suspected in the prostate (35, 36)

Uveitis (43)	Suspected	Mannan (21)	Associated with CD4+ T cell recognition of *Candida albicans* antigens (44). Mannan causes uveitis in animal model (45). Associated with chronic prostate inflammation (46, 47)

Multiple sclerosis (7)	Proposed here	*Candida* (27–29)	Dimethyl fumarate is effective (48), and has antifungal properties (49)

Systemic lupus erythematosus (50)	Not suspected	Mannan (22, 23)	

Goodpasture’s disease (51)	Not suspected	Not tested	

**Figure 1:**
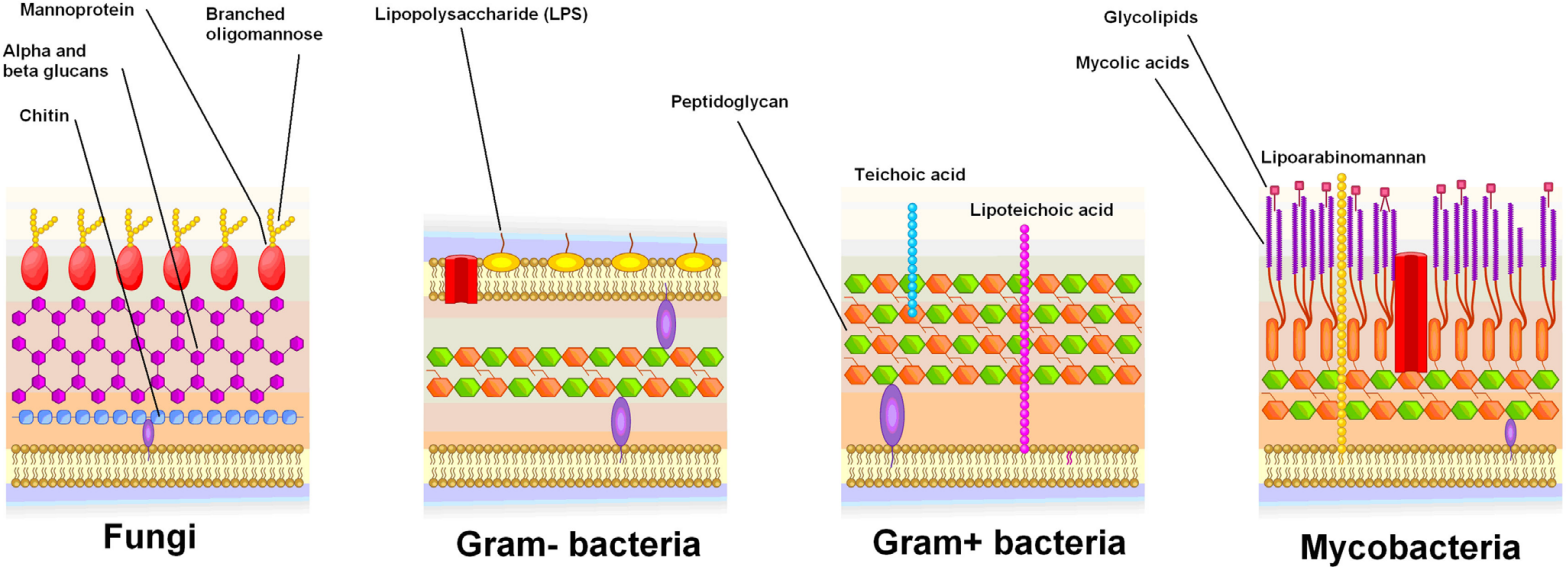
Cell wall structure of fungi and bacteria. Mannoproteins are specific to fungi, and are highly antigenic.

*Antibodies against mannan target mannose polymers covalently bound to fungal cell wall proteins (see mannoproteins in Figure 1); mannan is a minor component of mycobacteria cell walls, but is absent from other types of bacteria. Antibodies against beta-glucan target the cell wall of fungi (Figure 1); beta-glucan is also present in the cell wall of some types of bacteria*.

As first proposed for ABPA (30), the HLA-DRB1*15 allele group appears to increase immune sensitivity to fungi: this likely protects the host from certain infections, while sometimes causing chronic inflammation due to exposure to usually benign fungi such as *Aspergillus fumigatus*.

## Antibodies Against Fungi

HLA-DRB1*15 increases the risk of various conditions either caused or suspected of being caused by exposure to fungi (Table 1). This suggests MS risk might also be increased by exposure to fungi. All conditions in Table 1 that were studied for antibodies against fungi showed a positive association. This includes three studies which found that antibodies against various *Candida* species are associated with MS (27–29). With the exception of ABPA, it remains unclear how such antibodies can increase the risk of any of these diseases. Are memory B cells recognizing fungal epitopes directly involved, or are they a biomarker of another mechanism? The elimination of B cells in MS rapidly improves symptoms (52), suggesting they are directly involved, though the association with fungi remains speculative.

*Candida* species are the most common fungus on human mucosal surfaces, typically colonizing the host asymptomatically (53). Nearly half of adults have *Candida* in the mouth (53) or gut (14), and a majority of women have had genital exposure (54). Though the hypothesis of *Candida* infections reaching the CNS and directly causing MS has been proposed (27, 29), it appears unlikely that this would have gone unnoticed.

Antibodies against *Candida* indicate a past fungal infection produced memory B cells recognizing fungal epitopes. Memory B cells which recognize both a fungal epitope and an epitope in the CNS could explain the link between *Candida* antibodies and MS risk. As EBV infects naive B cells in mucosal surfaces exposed to fungi, “forbidden” memory B cells targeting fungal epitopes could be produced (55). Though speculative, this mechanism would explain three important observations. First, HLA-DRB1*15’s association with MS could be due to an increased probability of producing an immune response against fungi (Tables 1 and 2). Second, EBV’s association with MS could be due to naive B cells differentiating into “forbidden” memory B cells because they were exposed to both fungal antigens and EBV during affinity maturation (Table 3), resulting in the inadvertent recognition of an epitope in the CNS (55). Third, women’s elevated risk of MS (11) could be explained by higher antibody titers against *Candida albicans* measured in healthy women as compared to men, suggesting a higher probability of fungal infections in women perhaps due to genital exposure (56).

**Table 2:** Sites where HLA-DRB1*15 could be presenting fungal antigen peptides to CD4+ T cells, increasing the probability of memory B cells recognizing a “forbidden” epitope in the central nervous system (CNS).

Site	Presentation mechanism	Expected effects
Anywhere fungi is found in the body (e.g. mouth, genitals, gut, skin, lungs)	Antigen-presenting cells (e.g. macrophages and dendritic cells) endocytose whole fungi or fungal antigens, present fungal peptides to CD4+ T cells	CD4+ T cell population that recognizes fungal antigens expands. A large population of CD4+ T cells that recognize fungal peptides increases the probability of naive B cells loosely recognizing fungal antigens maturing into memory B cells
	Memory B cells whose B cell receptor (BCR) has high affinity to fungal antigens endocytose them, present fungal peptides to CD4+ T cells	Memory B cell population that recognizes fungal antigens expands. This may explain why a reduction in gut fungi affects inflammation elsewhere in the body

Lymph nodes	Resting naive B cells whose BCR loosely recognizes fungal antigens endocytose them, present fungal peptides to CD4+ T cells	CD4+ T cell recognition of fungal peptide allows naive B cells to activate and become B cell blasts, leading to somatic hypermutation and memory B cells with high affinity to fungal antigens. The association between Epstein-Barr virus (EBV) and multiple sclerosis (MS) suggests this mechanism is not important because EBV infects and activates resting naive B cells *without* CD4+ T cell help
	Maturing B cells whose BCR recognizes fungal antigens endocytose them, present fungal peptides to CD4+ (follicular helper) T cells	Increases probability of naive B cells maturing into memory B cells that recognize fungi

CNS	Memory B cells whose BCR has high affinity to a fungal epitope endocytose cognate antigens, present peptides to CD4+ T cells	Memory B cell activates in the CNS due to cognate antigens present in this site. Because CD4+ T cells do not seem to be necessary for active MS (52), memory B cells may be able to cause inflammation without CD4+ T cell help in the CNS

**Table 3:** Factors affecting the number of EBV infected memory B cells recognizing fungal antigens.

Factor	Mechanism	Expected effects
BCR of naive B cell	EBV virions infect resting naive B cells that have a “random” BCR. This “random” BCR is biased toward either fungal or bacterial antigens, and will thus bias affinity maturation	Variations in V(D)J genes and recombination during production of naive B cells in the bone marrow may be genetically biased toward either bacterial or fungal antigens, respectively reducing or increasing multiple sclerosis (MS) risk

Naive CD4+ T cell antigen recognition	Affinity maturation requires naive CD4+ T cells to clonally expand and become CD4+ (follicular helper) T cells. This requires dendritic cell presentation of antigens through HLA-D molecules, which will be biased toward antigens best recognized by naive CD4+ T cells	Variations in V(D)J genes and recombination during production of naive CD4+ T cells in the thymus may be genetically biased toward either bacterial or fungal antigens, respectively reducing or increasing MS risk. HLA-D molecules which efficiently present bacterial antigens should reduce MS risk, whereas HLA-D molecules which efficiently present fungal antigens should increase MS risk
CD4+ (follicular helper) T cell antigen recognition	Affinity maturation requires CD4+ (follicular helper) T cell recognition of a portion of the antigen endocytosed by the B cell through its BCR and presented through HLA-D molecules. During affinity maturation, competitive selection of somatically hypermutated B cells recognizing either bacterial or fungal antigens will be biased toward the antigen type best recognized by CD4+ (follicular helper) T cells	

Relative concentration of commensal microbe antigens in lymph node where EBV-positive naive B cells mature	The ratio of bacterial to fungal antigens in lymph nodes where EBV infected naive B cells mature will skew affinity maturation toward the more abundant antigen type due to improved receptor-ligand kinetics	A reduction of commensal bacteria (e.g. *Lactobacillus*) or an increase in commensal fungi (e.g. *Candida*) would increase MS risk. For example, antibacterial/antifungal drugs will dramatically increase and then decrease antigens in lymph nodes, due to the massive initial die-off of targeted microbes, followed by a suppression of the targeted microbial population

Total number of EBV infected B cells	CD8+ (cytotoxic) T cell control of the EBV infected B cell population determines how often a naive B cell matures into a memory B cell while EBV infected	Lower EBV loads result in fewer chances of producing a memory B cell which recognizes a “forbidden” antigen present in the central nervous system, in turn reducing MS risk. Individuals with strong CD8+ (cytotoxic) T cell control of EBV would be at lower MS risk. Interventions to lower the general B cell population (e.g. anti-CD20 drugs) or the EBV infected B cell population (e.g. using adoptive immunotherapy) would both be expected to reduce MS risk

*Commensal microbes collocated with Epstein-Barr virus (EBV) virions (e.g., in the mouth and genitals) provide an ideal antigenic target to complete EBV’s lifecycle, and are likely recognized by the B cell receptor (BCR) of EBV infected memory B cells. Bacteria are normally the dominant commensal microbe type in both sites, so EBV infected memory B cells would mostly target bacterial antigens. However, if sufficient fungal antigens are also present during affinity maturation, some EBV infected memory B cells’ BCRs may converge onto fungal rather than bacterial antigens. Note that an EBV-positive B cell that recognizes a “forbidden” antigen cannot do much harm immediately after leaving the germinal center: as long as a B cell remains EBV infected, CD8+ (cytotoxic) T cell control will prevent it from secreting antibodies or clonally expanding. Only after a few dozen divisions is the memory B cell expected to lose EBV episomes, and the associated CD8+ (cytotoxic) T cell control*.

## Other MS Biomarkers Consistent with a Fungal Etiology

### Mannoproteins

Mannoproteins are a ubiquitous component of fungal cell walls composed of protein-bound branched mannose polymers (Figure 1). Mammalian glycoproteins rarely have terminal mannose (57), making mannoproteins good antigens (58, 59). Innate immune recognition of mannoproteins by leukocytes occurs through macrophage mannose receptor (MMR) uptake, followed by presentation of mannoprotein peptides to CD4+ T cells using HLA-D molecules (58–60). As compared to cell walls of Gram-negative bacteria or *Saccharomyces cerevisiae* (a benign fungus), *C. albicans* mannoproteins induce strong production of IL-17 through this process (60). The proposed functions of the MMR include the clearance of fungi, bacteria, viruses, and homeostasis of human glycoproteins (61). The MMR is expressed by macrophages in active MS lesions, but not in controls or inactive disease (62). This suggests infectious agents *inside* the CNS may be contributing to inflammation in MS, or alternatively that human cell debris elicit MMR expression.

Mannose-binding lectin (MBL) is an innate immune defense protein synthesized in the liver, which binds to terminal mannose, *N*-acetylglucosamine, and fucose on microbial cell walls (63), and initiates the lectin complement pathway by activating mannose-binding protein-associated serine protease 2 (MASP-2) (64). MBL also binds to defective human glycoproteins lacking protective terminal residues such as galactose (57). Functional MBL/MASP-2 complexes are elevated in the plasma of MS cases as compared to controls, but not in the CSF (65). This suggests the immune response against infectious agents, such as bacteria or fungi, *outside* the CNS may be contributing to MS risk.

### IL-17

IL-17 is a cytokine produced by T cells (especially Th17 cells) and innate immune system cells (66) in response to intracellular and extracellular (67) bacterial and fungal infections (68, 69). For example, chronic *Helicobacter pylori* infections of the stomach induce IL-17 production (70). Genetic defects in the IL-17 pathway inhibit the clearance of fungal infections such as *C. albicans* (71).

IL-17 is also elevated in many idiopathic inflammatory diseases which do not have known infectious etiologies, such as psoriasis, ankylosing spondylitis, systemic lupus erythematosus, Crohn’s disease, and MS (72, 73). Thus IL-17 seems to play a role in chronic inflammation where infectious stimuli are lacking. Note that some of these conditions may have an as-yet-undiscovered infectious etiology, as became apparent for gastritis once *H. pylori* was discovered (74).

Though IL-17 has been implicated in the clearance of both bacterial and fungal infections (67, 75), its production is readily induced by *C. albicans* mannoproteins as compared to bacterial antigens (60). In a mouse model of arthritis stimulated by the injection of bacterial antigens into knee-joints, the addition of minute amounts of fungal antigens skewed the T cell immune response toward Th17 cells, tripling IL-17 concentration in the joints (76).

Though IL-17 is unambiguously associated with both fungal infections and MS, this association only provides weak circumstantial evidence of a causative link between MS and fungi, as IL-17 is also associated with bacterial infections and many idiopathic inflammatory diseases. Since IL-17 production is very sensitive to fungal antigens (60), even low fungal loads *inside* the CNS could provoke a Th17 immune response, especially in the presence of other inflammatory stimuli (76).

### Chitotriosidase

Chitotriosidase is a protein produced by activated macrophages, which hydrolyzes chitin (77). Chitin is a polysaccharide present in fungal cell walls, yet absent from bacterial and mammalian cells (Figure 1) (77). The exact function of chitotriosidase in the clearance of fungal infections is unclear (77). Macrophage chitotriosidase synthesis is stimulated by chitin, but not by other immunostimulatory compounds such as lipopolysaccharide or zymosan (78), suggesting that it is part of the innate immune response against chitin-bearing microbes (77).

Elevated chitotriosidase levels occur in a variety of diseases, both infectious and non-infectious (79). Chitotriosidase is thought to be a biomarker of macrophage activation, and may have functions unrelated to chitin (79). Elevated chitotriosidase in the CSF is an important biomarker of MS (80–82). This suggests a fungal infection *inside* the CNS may be contributing to inflammation in MS, or alternatively that macrophages produce chitotriosidase in response to non-chitin stimuli.

### Calprotectin

Calprotectin is a broad-spectrum antimicrobial protein complex produced mainly by neutrophils, but also by monocytes, macrophages, endothelial, and epithelial cells, which is released during infections or inflammation (83). Fungi are particularly susceptible to calprotectin, with minimum inhibitory concentrations of ~16 μg/ml as compared to 64–256 μg/ml for bacteria (84). Elevated calprotectin has been reported in many autoimmune diseases such as Crohn’s disease and systemic lupus erythematosus (85). Calprotectin levels in the CSF are markedly elevated during MS flare-ups (86), suggesting an infection may be present *inside* the CNS.

## Fungicides

Dimethyl fumarate (DMF) is an inexpensive fungicide (49) long used to protect consumer goods from fungi (87). In 2013, DMF was repurposed to treat MS in the United States of America (48). The New England Journal of Medicine nicknamed this the “Poison Chair” treatment, referring to its continued use as a fungicide on furniture, which sometimes causes cutaneous irritation (88). DMF had previously been approved in Germany to treat psoriasis (88). No consensus exists as to what its mechanisms of action are in MS or psoriasis. Anti-inflammatory interaction with the nuclear factor (erythroid-derived 2)-like 2 pathway has been proposed to explain the efficacy of DMF in reducing MS symptoms (89), though other immunomodulatory mechanisms continue to be investigated (90).

Several antifungal drugs have been shown to improve psoriasis symptoms: nystatin (20, 91–93), ketoconazole (94, 95), and itraconazole (96, 97). Elevated antibodies against *Malassezia furfur* (18, 19) and *C. albicans* (19) are risk factors of psoriasis, as is *Candida* colonization of the gut (14, 15). Cutaneous exposure to lysed *M. furfur* cells triggers psoriasis in susceptible individuals (98). These links between psoriasis and fungi suggest DMF’s antifungal properties may be important. The paucity of studies linking antifungal compounds other than DMF with a reduction in MS symptoms prevents a similar conclusion from being reached for MS: if future studies demonstrate the efficacy of many antifungal drugs in MS, this would provide strong evidence of a fungal etiology.

The effect of oral nystatin on psoriasis is surprising, as it is not absorbed (99), thus cannot reach fungi beyond the gut. Yet oral nystatin has been repeatedly shown to reduce psoriasis symptoms (20, 91–93), despite never reaching sites of psoriatic inflammation. Perhaps *Candida* colonization of the gut causes the proliferation of lymphocytes recognizing antigens on the skin (100, 101)—in other words an id reaction (102) stimulated by fungi in the gut. Unlike in psoriasis, the long-term use of oral nystatin has not been widely studied in MS. It would be interesting to know if it reduces MS symptoms as reported by Truss (26): this would suggest fungi *outside* the CNS are contributing to MS.

## An Example of Events Which may Lead to MS

The following example illustrates the four main hypothesized steps required to produce memory B cells recognizing an MS-causing antigen present in the CNS. They are based on our recent review of EBV and MS, which concluded that EBV coerces memory B cells to recognize commensal microbe antigens (55), resulting in “forbidden” memory B cells recognizing an antigen in the CNS, as originally proposed by Pender (103, 104). This process requires a memory B cell to encounter similar epitopes three times: first in the mouth (where the memory B cell originates), then anywhere in the body (where the memory B cell clonally expands), and finally in the CNS (where the memory B cell recognizes a “forbidden” antigen and induces MS-causing inflammation). The antigens, fungal species, and organs chosen in this example are for illustrative purposes only—the production of “forbidden” MS-causing memory B cells may occur in a number of different ways.

### EBV Produces “Forbidden” Memory B Cells in the Mouth

Benign commensal fungi frequently colonize the oral cavity and respiratory system, including *Aspergillus* species (105). *Aspergillus* cells are recognized by CD4+ T cells following HLA-D antigen presentation by phagocytes. This is particularly efficient in HLA-DRB1*1501 and HLA-DRB1*1503 carriers, and leads to a population of activated CD4+ T cells recognizing *Aspergillus* antigens in the lymph nodes of the mouth (Waldeyer’s ring).

Meanwhile, a resting naive B cell near Waldeyer’s ring is infected by an EBV virion: this occurs often in healthy EBV carriers (106). Once infected, this naive B cell migrates to a germinal center within Waldeyer’s ring, performs affinity maturation and emerges as an isotype-switched memory B cell containing a few dormant EBV episomes (106). EBV hides from the immune system within these long-lived memory B cells, maintaining a persistent infection for the rest of the host’s life (106). Eventually these memory B cells reactivate and differentiate into plasma cells in the mouth by finding a cognate antigen which is also recognized by CD4+ T cells (55, 106). EBV virions are then produced in these plasma cells, from which they can infect other hosts and maintain a stable EBV-positive memory B cell population in the same host (106).

Under EBV-free circumstances, a resting naive B cell only activates and goes through affinity maturation if its B cell receptor (BCR) has a decent affinity to an abundant antigen, and if it receives CD4+ T cell help for a peptide present in this antigen (106). When EBV infected, a naive B cell will activate and go through affinity maturation despite filling neither of these two conditions (106): its BCR will be honed to recognize any abundant antigen present in the lymph node (55). Which antigen is selected matters little for EBV, as long as the memory B cell eventually differentiates into a plasma cell in the mouth (55). This means commensal microbe antigens present in Waldeyer’s ring and recognized by CD4+ T cells in this site will be selected (55).

**Figure 2:**
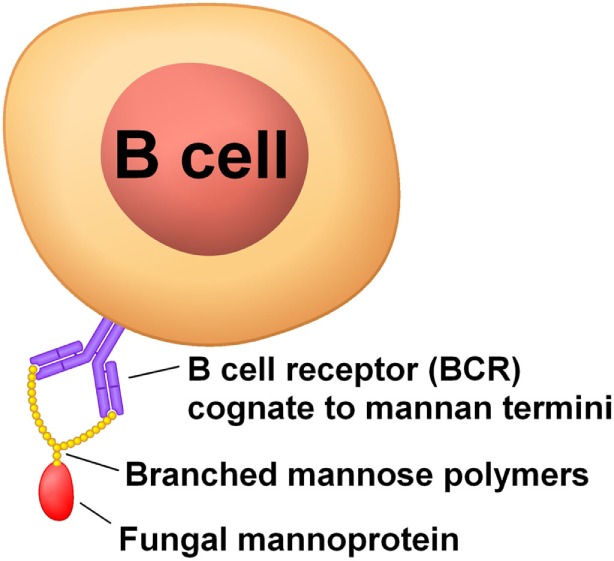
Mannoproteins have a high avidity to B cell BCRs because of the branched nature of their mannose polymers.

When present, CD4+ T cells recognizing *Aspergillus* antigens will skew epitope recognition of EBV-infected memory B cells toward fungal antigens by providing CD4+ T cell help during affinity selection in the light zone of the germinal center. Antibodies against the mannose polymer portion of fungal mannoproteins have been associated with many autoimmune diseases (Table 1), so these are good antigen candidates (Figure 1). Mannoproteins provide a high avidity target for memory B cells because of their branched mannose polymer structure: mannose termini attached to the same protein can simultaneously bind to each arm of a BCR (Figure 2). Their protein portion can be recognized by CD4+ T cells, giving memory B cells the CD4+ T cell help necessary to differentiate into a plasma cell in the mouth and shed EBV virions.

### “Forbidden” Memory B Cell Divides and Becomes EBV-Free

After completing affinity maturation, the EBV-infected memory B cell recirculates in peripheral blood and in the lymphatic system (106). During this time, no protein-coding genes from any of the EBV episomes in the memory B cell’s nucleus are expressed: this is essential for EBV’s survival because EBV-infected memory B cells are sought by a large number of T cells: ~3% of CD8+ T cells recognize EBV proteins (107).

In order to maintain long-term immunity, the memory B cell periodically divides—about 2.7% of memory B cells are dividing at any given time (106). During memory B cell division, EBV episomes must be replicated and partitioned between daughter cells. This is accomplished by expressing a single EBV gene (*EBNA-1*); the EBNA-1 protein is only present during cellular division to reduce the probability of the memory B cell being killed by CD8+ T cells while it is not dividing (106).

Unlike human chromosomes, EBV episome replication and partitioning is not very reliable, and after a few dozen divisions, some daughter cells become EBV-free (108). Once EBV-free, daughter memory B cells are no longer sought by T cells, so their longevity, clonal expansion, and differentiation into plasma cells cease being constrained by immune surveillance of EBV proteins.

### “Forbidden” Memory B Cell Clonally Expands in the Gut

When EBV first infects the host, up to half of memory B cells contain EBV episomes (106). This high viral load is due to a lack of T cells recognizing EBV proteins (106). As adaptive immunity is acquired over the following year, the fraction of memory B cells containing EBV episomes drops ten thousand-fold and stabilizes at about half a million memory B cells (or 0.001%) (106). For the rest of the host’s life, a small number of EBV-positive memory B cells are produced in the mouth to replace those eliminated by immune surveillance and other forms of attrition (106). A small subset of the EBV-positive memory B cell pool is expected to target a “forbidden” antigen and survive long enough to become EBV-free (55). However, this population of memory B cells is likely too small to cross the blood–brain barrier and find a cognate antigen in the CNS without first clonally expanding.

To clonally expand, the now EBV-free “forbidden” memory B cell that we have been following from the mouth must find a cognate antigen and an activated CD4+ T cell that recognizes a peptide from this antigen. Lymph nodes exposed to fungal antigens from the gastrointestinal tract and genitals provide the best environment for this chance event to occur—especially when these sites are colonized with *Candida*. CD4+ T cells which readily recognize fungal antigens should increase the probability of this chance event happening: such cells are associated with idiopathic uveitis (44), though no similar study could be found for MS. Antibodies against *Candida* species can be used as a proxy for CD4+ T cell sensitivity to fungal antigens: they are strongly associated with MS risk (27–29).

Fungal colonization is thus expected to be an MS risk factor because it allows “forbidden” memory B cells to clonally expand after shedding their EBV episomes. To the best of our knowledge, no study has directly measured fungal colonization in relation to MS. The efficacy of the oral fungicide DMF suggests fungal colonization of the gut may be an important contributor to the clonal expansion of “forbidden” MS-causing memory B cells.

### “Forbidden” Memory B Cell Recognizes an MS-Causing Antigen in the CNS

As the now large EBV-free “forbidden” memory B cell population recirculates through the blood and lymphatic system, a small fraction of these cells will reach the CNS. Once in the CNS, these cells will find an antigen cognate to their BCR, enabling the chain of events leading to demyelination. Eliminating these memory B cells should improve MS symptoms, as suggested by the efficacy of anti-CD20 drugs (52). The exact nature of the antigen found by “forbidden” memory B cells in the CNS is not known, though mannose polymers are plausible candidates.

Because fungi are eukaryotes, they have many genes which are similar to human genes: molecular mimicry due to an epitope conserved within opisthokonta could explain why memory B cells recognizing a fungal epitope also recognize a human epitope. Alternatively, there could be an elusive fungal infection in the CNS against which immune tolerance is lost in the presence of these memory B cells. Several histological reports are consistent with a fungus in the human brain, though definite proof is currently lacking (109–117).

The events in the example above account for the two best established MS risk factors: HLA-DRB1*15 and EBV seropositivity, as previously described (55). They also account for the association with antibodies against *Candida* (and thus female sex), and for the efficacy of DMF and anti-CD20 drugs in MS. However, they do not account for three other risk factors: age at onset (young adulthood), smoking, and herpes simplex virus type 2 (HSV-2) seropositivity (118–121). These three additional risk factors suggest a sexually acquired infection is involved.

## Genital Exposure to Fungi

Multiple sclerosis is rare in children, followed by a sudden increase in risk in adolescence (10). Nearly all types of infections (including EBV and fungi) are very common in children, except sexually transmitted infections (STIs). No known MS risk factors account for the low incidence of MS in children. If the set of infectious agents etiologically involved in MS does not include an STI, why is the rate of MS so low in childhood?

### Genital Exposure to *Candida*

While *Candida* is very common in children (53), vaginal *Candida* infections are rare in girls (54). The distribution of the age at first vulvovaginal candidiasis episode and MS onset match well (10, 54). Perhaps genital exposure to *Candida* species can trigger MS. Adult women have higher antibody titers against *Candida* than men, for which vulvovaginal candidiasis is the simplest explanation (56). This difference may contribute to women’s increased risk of MS as compared to men’s. Because antibodies against *Candida* are common in children (122), humoral immune responses to genital *Candida* infections cannot directly explain the very low incidence of MS in children. Only if the humoral immune responses in these sites were different, producing “forbidden” memory B cells in the genitals, but not in the mouth or gut, could vulvovaginal candidiasis explain the age at onset of MS. Perhaps naive B cells in genitalia are *simultaneously* exposed to EBV virions and *Candida* antigens, whereas combined exposure is rare in the mouth and gut. This hypothesis would be plausible for *Candida* in the gut (where EBV virions are generally absent), but not for *Candida* in the mouth (which is replete with EBV virions). While genital *Candida* exposure may well be an MS risk factor, it cannot account for the low incidence of MS in children.

### Could There Be an As-Yet-Unrecognized Sexually Acquired Fungal Infection?

Two diseases have low incidence in children, peak incidence in young adults, and moderate associations with smoking and HSV-2: cervical cancer, respectively OR = ~1.8 (123) and OR = ~1.6 (124) and MS, respectively OR = ~1.5 (8) and OR = ~1.6 (121). We now know that cervical cancer is not caused by HSV-2 and smoking—these two risk factors act as surrogates for oncogenic human papilloma virus (HPV) exposure, and their association with cervical cancer disappears after controlling for oncogenic HPV (124–128).

Multiple sclerosis’ association with young adulthood, smoking, and HSV-2 seropositivity remains unexplained. In 2002, Hawkes hypothesized that an as-yet-unrecognized STI was causing MS based on three observations (129). First, the distribution of the age at onset of common STIs approximately matches MS (10, 130). Second, MS “clusters” have been reported on small islands where soldiers were posted during World War II (129). Third, many known STIs disseminate throughout the body and cause chronic neurological symptoms through demyelination, such as syphilis (131), human T-lymphotropic virus (132), and human immunodeficiency virus (133). A fourth observation was added by Hawkes in 2006: HSV-2 seropositivity is associated with MS (121). We add a fifth observation: smoking increases MS risk, possibly by acting as a surrogate for an STI.

Though the existence of an as-yet-unrecognized STI may seem unlikely, this hypothesis has been proposed to explain the sexual risk factors of prostate cancer (134–136) (currently no strong candidate) (137) and reactive arthritis (138) (strongest candidate is *Chlamydia trachomatis*, found in ~13% of cases and ~2.4% of controls) (139). Prostate cancer has recently been linked to an intracellular fungal infection (35, 36). Antibodies against fungi have been linked to reactive arthritis symptoms, especially uveitis (21). Older studies have found antibodies against a component of the prostate and prostatic inflammation in reactive arthritis (140–143) and uveitis (46, 47, 144) patients. Finally, HLA-DRB1*15 and antibodies against a component of the prostate have been linked to granulomatous prostatitis (42, 145).

Together, these studies suggest genital exposure to fungi may play an important etiological role in prostate disease, reactive arthritis, and MS. Could the unexplained histological evidence of fungal cells in the CNS (110, 111, 113, 117) and in the prostate (146, 147) be caused by a fungal infection spreading from the genitals after sexual debut? If so, loss of immune tolerance to this putative infection could be a plausible cause of prostate disease, reactive arthritis, uveitis, and MS.

## Conclusion

Varied molecular and epidemiological evidence supports a role for infections in MS (Table 4). EBV is the most strongly associated infection, though the underlying mechanisms are not firmly established (55). Unlike EBV (148), MS is rare in children (10), suggesting EBV is insufficient to cause MS on its own. Genital exposure to an infection involved in MS could solve the puzzling lag between childhood EBV infections and MS onset (129).

**Table 4:** Summary of findings linking multiple sclerosis (MS) to various infection types and recognition of self-antigens.

Finding	Finding strength	Suggests contributing microbe is located:	Fungi	Bacteria	Viruses	Self-antigens
Epstein-Barr virus (EBV) necessary for most MS cases (4–6, 55)	++++	See Table 3	+	+	EBV: +++	+/−
					Others: −	

HLA-DRB1*15 increases risk of MS (7)	++++	See Table 2	+	+	+	+/−
	
HLA-DRB1*15 increases immune response to fungi (see Table 1)	+++		+	−	−	+/−

B cells necessary for active MS (52)	+++		+	+	+	+

CD4+ T cells not necessary for active MS (52)	+++	Outside central nervous system (CNS)	+	+	+	+

Oral antifungal dimethyl fumarate (DMF) reduces MS symptoms (48)	+++	In gut	+	+/−	+/−	+/−

Age at onset matches sexual debut (10)	+++	In genitals	++	++	++	+/−
Antibodies against herpes simplex virus type 2 associated with MS (118–121)	+++					
Smoking increases MS risk (8)	+++					

Chitotriosidase (*CHIT1*) elevated in MS (80–82)	++	In CNS	+	+/−	+/−	+/−

Antibodies against *Candida* associated with MS (27–29)	++		+	−	−	+/−

IL-17 elevated in MS (72, 73)	++	In CNS	+	+	+/−	+/−

Fungal antigens strongly induce IL-17 (60, 76)	++		+	+/−	+/−	+/−

Macrophage mannose receptor (MMR) in active MS lesion macrophages (62)	+	In CNS	+	+	+	+/−

Plasma functional mannose-binding lectin/mannose-binding protein-associated serine protease 2 complex elevated in MS, but unchanged in the cerebrospinal fluid (65)	+	Outside CNS	++	++	++	+/−

Calprotectin elevated in MS relapse (86)	+	In CNS	+	+	+/−	+/−

Oral antifungal nystatin reduces MS symptoms (26)	+/−	In gut	+++	−	−	+/−

*Finding strength is based on the number of published studies that strongly support the finding*.

The links between various idiopathic inflammatory conditions and antibodies against fungi (Table 1) could be explained by memory B cells accidentally recognizing benign antigens due to the simultaneous presence of EBV virions and fungal antigens near the same lymph node (55). Since fungi are eukaryotes, cross-reactive antibodies between fungal and human proteins are plausible. Alternatively, an elusive fungal infection could be present in the CNS, though there is currently no strong evidence supporting this. Excluding the presence of non-abundant fungi in the CNS is not trivial: the Th17 immune response is extremely sensitive to fungal antigens, suggesting even minute quantities could result in a robust response (60, 76).

Evidence presented here is also consistent with pathways *shared* between the immune response to fungi and MS inflammation, which means links with fungi could be coincidental. To establish causality, either many antifungal drugs must be shown to improve MS symptoms or case-control studies must show a strong association with specific fungal species. Neither condition is met today.

Thorough microbiome studies of the CNS, mouth, gut, and genitalia in association with MS should be run. Particular attention should be given to fungal infections which generally do not affect children, such as vulvovaginal candidiasis. A very recent study strongly suggests many microbes present in humans have yet to be discovered (149). Older molecular surveys of oral (105) and genital (150) fungi in healthy individuals found many novel species: the most common fungus found in the vagina was not a *Candida* species as expected, but rather an unknown species detected in about 25% of healthy individuals (150).

## Author Contributions

The authors both contributed in drafting and reviewing the article.

## Conflict of Interest Statement

The authors declare that the research was conducted in the absence of any commercial or financial relationships that could be construed as a potential conflict of interest.
